# Levodopa-Induced Dyskinesia Is Related to Indirect Pathway Medium Spiny Neuron Excitotoxicity: A Hypothesis Based on an Unexpected Finding

**DOI:** 10.1155/2016/6461907

**Published:** 2016-04-06

**Authors:** Svetlana A. Ivanova, Anton J. M. Loonen

**Affiliations:** ^1^Mental Health Research Institute, Tomsk, Russia; ^2^National Research Tomsk Polytechnic University, Tomsk, Russia; ^3^Department of Pharmacy, University of Groningen, Groningen, Netherlands; ^4^GGZ Westelijk Noord-Brabant, Bergen op Zoom, Netherlands

## Abstract

A serendipitous pharmacogenetic finding links the vulnerability to developing levodopa-induced dyskinesia to the age of onset of Huntington's disease. Huntington's disease is caused by a polyglutamate expansion of the protein huntingtin. Aberrant huntingtin is less capable of binding to a member of membrane-associated guanylate kinase family (MAGUKs): postsynaptic density- (PSD-) 95. This leaves more PSD-95 available to stabilize NR2B subunit carrying NMDA receptors in the synaptic membrane. This results in increased excitotoxicity for which particularly striatal medium spiny neurons from the indirect extrapyramidal pathway are sensitive. In Parkinson's disease the sensitivity for excitotoxicity is related to increased oxidative stress due to genetically determined abnormal metabolism of dopamine or related products. This probably also increases the sensitivity of medium spiny neurons for exogenous levodopa. Particularly the combination of increased oxidative stress due to aberrant dopamine metabolism, increased vulnerability to NMDA induced excitotoxicity, and the particular sensitivity of indirect pathway medium spiny neurons for this excitotoxicity may explain the observed increased prevalence of levodopa-induced dyskinesia.

## 1. Introduction

Over 45 years after discovery of its beneficial effects [1, 2], levodopa remains the mainstay of treatment of Parkinson's disease (PD). Usage of levodopa for treatment of motor symptoms at all stages of PD is supported by strong evidence [3]. However, long-term levodopa treatment of PD is frequently complicated by motor fluctuations and dyskinesia [3–6]. Monotherapy with dopamine agonists in the early phases of the disease reduces the risk of dyskinesia compared with levodopa, but dopamine agonists are unable to prevent dyskinesia once levodopa is added, which is always required once disease severity progresses [7]. Until now, no effective drug treatment of levodopa-induced dyskinesia (LID) has been developed [8]. Several theories try to explain the pathophysiology of this important treatment complication with the ultimate goal of developing such treatments [8–10]. However, the exact pathological mechanism has also not yet been elucidated.

Recently, our group has published our serendipitous finding in a small group of patients we used as a control group in a study on tardive dyskinesia (TD) [11, 12]. Our analysis revealed that two* GRIN2A* variants, rs7192557 and rs8057394, were most frequently associated with dyskinesia in our control group of patients with neurological disorders (101 with Parkinson's disease, 21 with essential tremor, and 21 with different forms of dystonia). It is important to notice that rs7192557 is a tagging SNP for rs1969060, which was previously associated with the age of onset in Huntington's disease, and that SNP rs8057394 was the other variant identified in this study of Arning et al. [13]. In our study we found that rs7192557 and rs8057394 were not significantly associated with tardive dyskinesia in 431 patients with schizophrenia [11].

Huntington's disease (HD) is an inherited neurodegenerative disorder, characterized by progressively worsening chorea, cognitive and psychiatric disturbances involving the basal ganglia and cerebral cortex [14]. HD is caused by a mutation of the gene of huntingtin protein (htt), located in the 4p16.3 region of the short arm of chromosome 4, showing an increased number of CAG nucleotides [15]. The involuntary movement disorder in HD [16] is similar to that of LID [4], although in both cases symptomatology is highly variable and in HD dependent on disease stage. In HD, the disease process has been linked to NMDA receptor-induced excitotoxicity in the striatal medium-sized spiny neurons (MSNs) that form the indirect pathway [14, 15, 17].

In the current paper we try to examine a possible similarity between the pathological mechanisms of LID and HD based on the found association of two* GRIN2A* variants and the progression of both diseases. We start with a description of the structure and function of the NMDA receptor and its disfunctioning in HD.

## 2. Glutamatergic NMDA Receptor Neurotoxicity

The* GRIN2A* gene encodes for a protein which is part of the ionotropic glutamatergic N-methyl-D-aspartate (NMDA) receptor [18]. The amino acid L-glutamate is an excitatory neurotransmitter in the central nervous system, mediating its effects via ionotropic and metabotropic receptors. Ionotropic receptors are ligand-gated channels and divided into three groups, one of which is the group of NMDA receptors. These NMDA receptors consist of four (or five) proteins belonging to three different types: NR1, NR2, or NR3. At least one NR1 subunit is present in the NMDA receptor. This NR1 subunit combines with at least one modulatory NR2 subunit and more infrequently a modulatory NR3 subunit. Four different NR2 subunits have been identified: NR2A, NR2B, NR2C, and NR2D. Together, these complexes form membrane-bound, nonselective cation channels characterized by voltage-dependent activity, high calcium permeability, and comparatively slow activation/deactivation kinetics [18].

NR2A and NR2B are relatively large proteins with molecular masses of about 170–180 kDa. They have a large extracellular N-terminal region, which carries a glutamate binding site, three relatively small transmembrane domains (M1, M3, and M4), and a small reentry loop (M2), which is critical for Ca^2+^ permeability and Mg^2+^ sensitivity [18]. NMDA receptors are anchored within the postsynaptic surface membrane by binding through their C-terminal region to synaptic proteins associated with the membrane cytoskeleton. An example is the binding of NR2A and NR2B subunits to members of the membrane-associated guanylate kinase family (MAGUKs): postsynaptic density- (PSD-) 95/synapse associated protein- (SAP-) 90 [19]. Binding to MAGUKs links NMDA receptors to other membrane proteins on one hand and to important intracellular signaling proteins on the other [20]. Binding to MAGUKs also plays an important role in trafficking NMDA receptors between synaptic and extrasynaptic (intracellular) localization [20, 21]. Changing these signaling and trafficking activities may play a role in NMDA associated excitotoxicity in HD. Sun et al. provided evidence that normal huntingtin is associated with N-methyl-D-aspartate (NMDA) receptors via postsynaptic density-95 (PSD-95) and that polyglutamine expansion interferes with the ability of huntingtin to interact with PSD-95 [22]. Fan et al. provided direct evidence that polyglutamate expansion of huntingtin increases the interaction between NR2B and PSD-95 [23]. This results in increased NMDA receptor activity as is, for example, reflected by the evoked current amplitude and caspase-3 activity, an enzyme playing a central role in regulating neuronal death in the CNS [24]. This effect appears to be selective for NMDA receptors carrying NR2B subunits, as NR2A-NMDA receptors showed similar sensitivity when coexpressed with mutant versus normal huntingtin [25].

In spite of this selectivity of huntingtin protein for affecting neurotoxicity of NR2B carrying NMDA receptors, also NR2A subunits have been associated with modulation of this neurotoxicity. However, the results are not entirely consistent. Arning et al. described that in a cohort of 167 HD patients a significant part of the variance of age of onset of illness could be attributed to a GRIN2B (rs1806201) and to a lesser extent to a GRIN2A (rs1969060) variant [26]. These authors later fine-mapped a larger HD sample (*N* = 250) and showed that two additional SNPs (rs8057394 and rs2650427) showed an even stronger association than the originally identified GRIN2A variant [13]. Andresen et al. tried to confirm these results in 443 subjects who were heterozygotes for the HD gene (only one allele with >35 CAG repeats) and had been diagnosed with Huntington's disease [27]. In this Venezuelan sample again evidence was found of association of the GRIN2A SNP (rs1969060), but not of the GRIN2B variant. In a European cohort of 1,069 HD patients the analysis did not, however, replicate the association between the SNP rs1969060 and the age of onset [28]. However, in 241 patients initially presenting psychiatric symptoms rs1969060 polymorphisms explained a considerable amount of variance in residual age of onset. The GRIN2A SNP rs2650427 showed a significant association with the age of onset of symptoms in all 1,069 patients. Ramos et al. studied 1,619 HD patients and found no evidence for a significant association between GRIN2A SNP rs2650427 polymorphisms and the age of onset of motor symptoms [29].

This type of differences is not very surprising. Variations of the GRIN2A genotype probably only determine the vulnerability to developing HD symptoms to a minor degree and there may exist several factors which contribute to a similar degree. The heterozygosity may vary greatly between various patient populations. As a matter of fact, the C allele frequency of the GRIN2A C/T SNP (rs2650427) is 17% in Europe and 52–56% in Asia and Africa [27]. Moreover, especially in large patients cohorts, the results of association studies may be flawed by poor phenotyping and the contribution of numerous, not accounted for, confounders. In addition, both SNPs rs1969060 and rs2650427 are positioned in intron parts of the* GRIN2A* gene on chromosome 16 [13]. Therefore, these variations cannot directly result in a change of NR2A function. However, the found associations support the idea that modification of the efficacy of NR2A containing NMDA receptors may have consequences for the likelihood of the excitotoxicity promoted by HD related changes in huntingtin protein.

**Figure 1:**
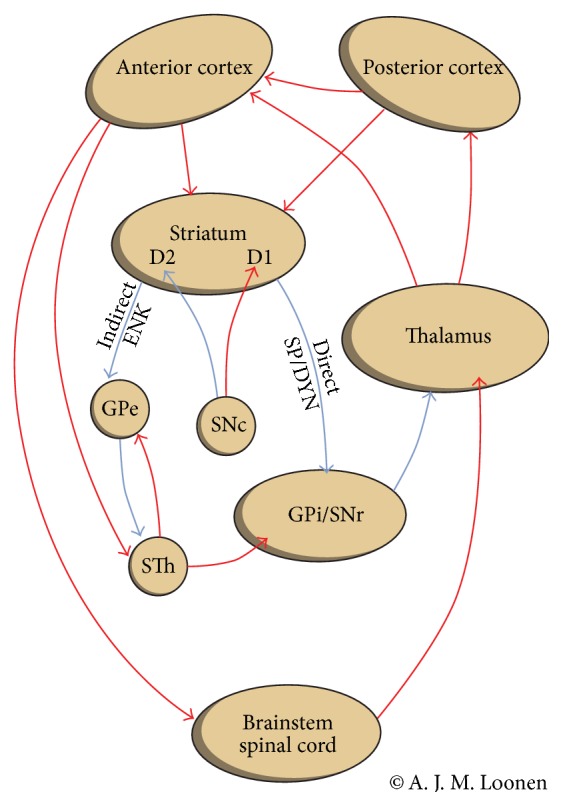
The cortico-striatal-thalamic-cortical circuits, including the indirect and direct pathways; ENK = enkephalin; GPe = globus pallidus, external segment; GPi = globus pallidus, internal segment; SNc = substantia nigra, pars compacta; SNr = substantia nigra, pars reticulata; SP/DYN = substance P/dynorphin; STh = subthalamic nucleus; D1 or D2: medium-sized spiny neurons with D1 or D2 receptors.

Glutamate is the most ubiquitous excitatory neurotransmitter in the nervous system, but in HD indirect pathway medium spiny neurons are primarily affected (Figure 1). This can be explained by looking at the distribution of NMDA receptor subunits. Although NR1 is widely distributed throughout most neurons in the brain, each of the NR2 subunits shows a more discrete distribution. NR2A and NR2B are highly expressed in most neurons of the cerebral cortex, hippocampus, and thalamus, whereas NR2C is mainly expressed in the cerebellum (where NR2A, but not NR2B, may also be found) and NR2D in the brainstem and spinal cord [30, 31]. NR2B subunits have been shown to be more extensively expressed relative to NR2A in the rodent striatum [31, 32]. This is used to explain the vulnerability of striatal medium spiny neurons to polyglutamate huntingtin [32]. We are not aware of experiments showing differences of the distribution of NR2A and NR2B subunits between MSNs of the direct and indirect pathway. However, striatal neuronal subtypes are selectively protected by neurotrophic factors against excitotoxic insults [33]. This makes MSN of the indirect pathway far more vulnerable than those of the direct pathway [33].

## 3. Mechanism of Levodopa-Induced Dyskinesia

The mechanism of levodopa-induced dyskinesia is usually studied in animal models in which nigrostriatal dopaminergic neurons have been lesioned with 1-methyl-4-phenyl-1,2,3,6-tetrahydropyridine (MPTP) or 6-hydroxydopamine (6-OHDA) [4, 8, 34]. In our opinion these models have a serious limitation, namely, that these animals do not suffer from genuine PD. In PD the degeneration of nigrostriatal dopaminergic neurons has to be attributed to genetically determined metabolic alterations which increase the neurotoxicity of dopamine and/or metabolites. These alterations occur within dopaminergic neurons during the initial disease process, but it is very unlikely that exogenous levodopa will be processed differently. Therefore, patients with genuine PD must be far more vulnerable to these effects than MPTP- or 6-OHDA-lesioned animals. Therefore, animal models can better be considered to indicate how levodopa acutely induces dyskinesia within nigrostriatal dopamine-deficient animals instead of models for the pathophysiological mechanism of LID. Dopamine can be considered to be a particularly neurotoxic endogenous substance. Dopamine metabolism results in releasing hydrogen peroxide, which results in the production of free radicals, which then cause cell damage [35]. This damage is limited by the influence of certain enzymes, like manganese superoxide dismutase (MnSOD), which scavenge these free radicals. This neurotoxicity is potentiated by a high level of activation of NMDA receptors, which also increases oxidative stress by finally activating nitric oxide synthase (nNOS) [18].

We want to hypothesize that the pathological basis of LID might be degeneration of indirect pathway medium spiny neurons. This could best be studied by comparing the striatal neuronal composition of patients with PD with and without dyskinesia and that of normal controls. Unfortunately, we did not find such comparisons described in the literature. Calon et al. found increased binding to NR1/NR2B and AMPA glutamate receptors in the lateral putamen associated with the clinical observation of LD-induced motor complications in advanced PD, but this tells relatively little about their subcellular localization and the mechanism behind it [36].

**Figure 2:**
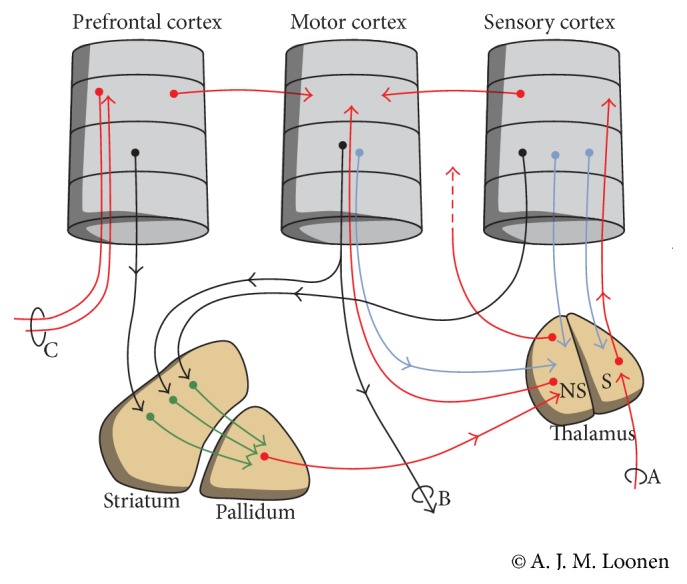
Simplified representation of the corticostriatal processing unit in which cortical information leading to a movement is processed in an intracortical and (parallel) extrapyramidal fashion; (A) sensory input, (B) projections to brainstem and spinal cord, and (C) projection to and from ipsilateral and contralateral cortical areas. NS = nonspecific part; S = specific part.

When our hypothesis is accepted, why the clinical presentation of LID is so variable becomes better understandable. The pattern of dyskinesia varies with respect to the time of onset in relation to levodopa intake [8]. This might result from instability of the system due to preferential degeneration of indirect pathway MSN in comparison to GABAergic projection neurons of the direct pathway. A classical theory of the mechanism of LID tells that, due to the degeneration of dopaminergic terminals, the “buffering capacity” of presynaptic uptake has disappeared [8]. Therefore, exogenous levodopa results in pulsatile under- and overstimulation of dopaminergic receptors [4, 8]. Chronic intermittent stimulation of normally tonically active dopaminergic D1 receptors brings about alterations in cell signals in corticostriatal and thalamostriatal glutamatergic synapses. This causes long-term potentiation of these excitatory glutamatergic efferents, particularly, resulting in overstimulation of direct pathway medium spiny neurons [4]. However, we want to emphasize that these changes will have a far larger impact when the regulatory system is already unstable. It should be kept in mind that the extrapyramidal circuit is a sophisticated regulatory system which controls the output of frontal cortical areas by converging direct and indirect pathways coming from and connecting several other cortical domains (Figures 1 and 2) [12]. Most activation patterns are learned during training the execution of specific movement complexes. This process is probably more easily disturbed by adverse long-term potentiation of corticostriatal and thalamostriatal synapses when the integrity of the system itself is compromised.

Why would a change within the functioning of NMDA receptors carrying NR2A subunits result in increased vulnerability to develop LID, as we have found in our Siberian PD patients [11]? This was not a tiny effect. For example, the odds ratio for an association between rs7192557 (tagging rs1969060) and LID was 3.21 (95% CI: 1.37–7.51, *p* = 0.0062) for 101 patients diagnosed with Parkinson's disease and 2.71 (95% CI: 1.35–5.46, *p* = 0.0046) for the whole group of 143 patients with neurological disorders. For rs8057394, these figures were 3.59 (95% CI: 1.48–8.71, *p* = 0.0033) and 2.86 (95% CI: 1.42–5.76, *p* = 0.0028), respectively. In the same study we found no significant association at all between these two SNPs and tardive dyskinesia in 431 patients with schizophrenia (*N* = 401, 95.1%) or schizotypical disorders according to ICD-10 criteria. This might be related to the specific genetic makeup of patients suffering from PD. Schizophrenic patients do not have a genetically determined vulnerability of dopaminergic neurotoxicity which is needed to become specifically vulnerable to this NMDA effect.

## 4. Conclusions

We want to hypothesize that NMDA receptor related excitotoxicity plays an important role in both HD and LID. In HD, excitotoxicity is caused by disinhibition of NR2B carrying NMDA receptors which are more extensively stabilized within the synaptic membrane and therefore are regularly too active (become excitotoxic). In LID, we suggest that the vulnerability to normal excitotoxic insults is potentiated due to the contribution of increased intracellular oxidative stress. This last phenomenon is expected to be caused by the same genetic composition which is causing degeneration of dopaminergic nigrostriatal neurons in these patients. As medium spiny neurons of the indirect pathway are more vulnerable to excitotoxicity than those of the direct pathway, a mismatch between their activities is caused, which results in instability and therefore an increased chance of adverse reactions to stimulation of dopamine receptors.

## References

[1] Birkmayer W., Hornykiewicz O. (1961). The L-3,4-dioxyphenylalanine (DOPA)-effect in Parkinson-akinesia. *Wiener klinische Wochenschrift*.

[2] Hornykiewicz O. (2010). A brief history of levodopa. *Journal of Neurology*.

[3] Connolly B. S., Lang A. E. (2014). Pharmacological treatment of Parkinson disease. A review. *The Journal of the American Medical Association*.

[4] Thanvi B. R., Lo T. C. N. (2004). Long term motor complications of levodopa: clinical features, mechanisms, and management strategies. *Postgraduate Medical Journal*.

[5] Thanvi B., Lo N., Robinson T. (2007). Levodopa-induced dyskinesia in Parkinson's disease: clinical features, pathogenesis, prevention and treatment. *Postgraduate Medical Journal*.

[6] Del Sorbo F., Albanese A. (2008). Levodopa-induced dyskinesias and their management. *Journal of Neurology*.

[7] Pilleri M., Antonini A. (2015). Therapeutic strategies to prevent and manage dyskinesias in Parkinson's disease. *Expert Opinion on Drug Safety*.

[8] Huot P., Johnston T. H., Koprich J. B., Fox S. H., Brotchie J. M. (2013). The pharmacology of L-DOPA-induced dyskinesia in Parkinson's disease. *Pharmacological Reviews*.

[9] Bargiotas P., Konitsiotis S. (2013). Levodopa-induced dyskinesias in Parkinson's disease: emerging treatments. *Neuropsychiatric Disease and Treatment*.

[10] Cerasa A., Fasano A., Morgante F., Koch G., Quattrone A. (2014). Maladaptive plasticity in levodopa-induced dyskinesias and tardive dyskinesias: old and new insights on the effects of dopamine receptor pharmacology. *Frontiers in Neurology*.

[11] Ivanova S. A., Loonen A. J. M., Pechlivanoglou P. (2012). NMDA receptor genotypes associated with the vulnerability to develop dyskinesia. *Translational Psychiatry*.

[12] Loonen A. J. M., Ivanova S. A. (2013). New insights into the mechanism of drug-induced dyskinesia. *CNS Spectrums*.

[13] Arning L., Saft C., Wieczorek S., Andrich J., Kraus P. H., Epplen J. T. (2007). NR2A and NR2B receptor gene variations modify age at onset in Huntington disease in a sex-specific manner. *Human Genetics*.

[14] Kumar P., Kalonia H., Kumar A. (2010). Huntington's disease: pathogenesis to animal models. *Pharmacological Reports*.

[15] Estrada Sánchez A. M., Mejía-Toiber J., Massieu L. (2008). Excitotoxic neuronal death and the pathogenesis of Huntington's disease. *Archives of Medical Research*.

[16] Sturrock A., Leavitt B. R. (2010). The clinical and genetic features of Huntington disease. *Journal of Geriatric Psychiatry and Neurology*.

[17] Fan M. M. Y., Raymond L. A. (2007). N-Methyl-d-aspartate (NMDA) receptor function and excitotoxicity in Huntington's disease. *Progress in Neurobiology*.

[18] Chaffey H., Chazot P. L. (2008). NMDA receptor subtypes: structure, function and therapeutics. *Current Anaesthesia and Critical Care*.

[19] Niethammer M., Kim E., Sheng M. (1996). Interaction between the C terminus of NMDA receptor subunits and multiple members of the PSD-95 family of membrane-associated guanylate kinases. *Journal of Neuroscience*.

[20] van Zundert B., Yoshii A., Constantine-Paton M. (2004). Receptor compartmentalization and trafficking at glutamate synapses: a developmental proposal. *Trends in Neurosciences*.

[21] Prybylowski K., Wenthold R. J. (2004). N-methyl-D-aspartate receptors: subunit assembly and trafficking to the synapse. *Journal of Biological Chemistry*.

[22] Sun Y., Savanenin A., Reddy P. H., Liu Y. F. (2001). Polyglutamine-expanded huntingtin promotes sensitization of *N*-methyl-D-aspartate receptors via post-synaptic density 95. *The Journal of Biological Chemistry*.

[23] Fan J., Cowan C. M., Zhang L. Y. J., Hayden M. R., Raymond L. A. (2009). Interaction of postsynaptic density protein-95 with NMDA receptors influences excitotoxicity in the yeast artificial chromosome mouse model of Huntington's disease. *Journal of Neuroscience*.

[24] Zeron M. M., Hansson O., Chen N. (2002). Increased sensitivity to N-methyl-D-aspartate receptor-mediated excitotoxicity in a mouse model of Huntington's disease. *Neuron*.

[25] Chen N., Luo T., Wellington C. (1999). Subtype-specific enhancement of NMDA receptor currents by mutant huntingtin. *Journal of Neurochemistry*.

[26] Arning L., Kraus P. H., Valentin S., Saft C., Andrich J., Epplen J. T. (2005). *NR2A* and *NR2B* receptor gene variations modify age at onset in Huntington disease. *Neurogenetics*.

[27] Andresen J. M., Gayán J., Cherny S. S. (2007). Replication of twelve association studies for Huntington's disease residual age of onset in large Venezuelan kindreds. *Journal of Medical Genetics*.

[28] Saft C., Epplen J. T., Wieczorek S. (2011). NMDA receptor gene variations as modifiers in Huntington disease: a replication study. *PLoS Currents*.

[29] Ramos E. M., Latourelle J. C., Gillis T. (2013). Candidate glutamatergic and dopaminergic pathway gene variants do not influence Huntington's disease motor onset. *Neurogenetics*.

[30] Hollmann M., Heinemann S. (1994). Cloned glutamate receptors. *Annual Review of Neuroscience*.

[31] Li L., Fan M., Icton C. D. (2003). Role of NR2B-type NMDA receptors in selective neurodegeneration in Huntington disease. *Neurobiology of Aging*.

[32] Raymond L. A. (2003). Excitotoxicity in Huntington disease. *Clinical Neuroscience Research*.

[33] Rikani A. A., Choudhry Z., Choudhry A. M. (2014). The mechanism of degeeeration of striatal neuronal subtypes in Huntington disease. *Annals of Neurosciences*.

[34] Fisone G., Bezard E. (2011). Molecular mechanisms of L-dopa-induced dyskinesia. *International Review of Neurobiology*.

[35] Lohr J. B., Kuczenski R., Niculescu A. B. (2003). Oxidative mechanisms and tardive dyskinesia. *CNS Drugs*.

[36] Calon F., Rajput A. H., Hornykiewicz O., Bédard P. J., Di Paolo T. (2003). Levodopa-induced motor complications are associated with alterations of glutamate receptors in Parkinson's disease. *Neurobiology of Disease*.

